# Borrowing From Nature: Biopolymers and Biocomposites as Smart Wound Care Materials

**DOI:** 10.3389/fbioe.2018.00137

**Published:** 2018-10-02

**Authors:** Giulia Suarato, Rosalia Bertorelli, Athanassia Athanassiou

**Affiliations:** ^1^Smart Materials, Istituto Italiano di Tecnologia, Genoa, Italy; ^2^In vivo Pharmacology Facility, Istituto Italiano di Tecnologia, Genoa, Italy

**Keywords:** wound healing, biomimetic, alginate, chitosan, hyaluronic acid, silk fibroin, keratin, antibacterial

## Abstract

Wound repair is a complex and tightly regulated physiological process, involving the activation of various cell types throughout each subsequent step (homeostasis, inflammation, proliferation, and tissue remodeling). Any impairment within the correct sequence of the healing events could lead to chronic wounds, with potential effects on the patience quality of life, and consequent fallouts on the wound care management. Nature itself can be of inspiration for the development of fully biodegradable materials, presenting enhanced bioactive potentialities, and sustainability. Naturally-derived biopolymers are nowadays considered *smart materials*. They provide a versatile and tunable platform to design the appropriate extracellular matrix able to support tissue regeneration, while contrasting the onset of adverse events. In the past decades, fabrication of bioactive materials based on natural polymers, either of protein derivation or polysaccharide-based, has been extensively exploited to tackle wound-healing related problematics. However, in today's World the exclusive use of such materials is becoming an urgent challenge, to meet the demand of environmentally sustainable technologies to support our future needs, including applications in the fields of healthcare and wound management. In the following, we will briefly introduce the main physico-chemical and biological properties of some protein-based biopolymers and some naturally-derived polysaccharides. Moreover, we will present some of the recent technological processing and green fabrication approaches of novel composite materials based on these biopolymers, with particular attention on their applications in the skin tissue repair field. Lastly, we will highlight promising future perspectives for the development of a new generation of environmentally-friendly, naturally-derived, smart wound dressings.

## Introduction

Skin is our major external defense system, in charge of protecting our inner body structures from microorganisms' attacks, and the adverse effects of the external environment. Adult skin is composed of three layers: *epidermis* or *stratum corneum*, mainly consisting of keratinocytes; *dermis*, the connective tissue rich in collagen; and *hypodermis* or *subcutaneous layer*, composed of fat tissue, which provides thermal isolation and mechanical protection to the body (Gurtner et al., 2008). Wounds are breaks or defects within the skin, which may form due to physicochemical or thermal damage. Acute wounds define injured tissues that need a healing period over 8–12 weeks, (e.g., burns, chemical injuries, cuts). In contrast, chronic wounds are a fallout of diseases, such as venous or arterial vascular insufficiency, pressure necrosis, cancer, and diabetes (Sen et al., 2009; Moura et al., 2013). They require longer healing time (weeks-months to years) and often fail to reach a normal healthy state, persisting in a pathological condition of inflammation (Guo and Dipietro, 2010). Therefore, delayed or impaired wound healing poses a significant socio-economic burden on patients and health care systems worldwide, in terms of treatment costs and waste production (Sen et al., 2009).

Insight into the intricate biochemical events activated during skin repair is crucial to design appropriate wound dressings (Weller and Sussman, 2006; Gurtner et al., 2008; Pereira et al., 2013). The healing process can be divided into the following, overlapping stages: homeostasis, inflammation, proliferation, and remodeling (Martin, 1997; Gurtner et al., 2008; Bielefeld et al., 2013; Das and Baker, 2016). *Homeostasis* is the immediate response of the body to an injury, in order to stop blood loss at the wound site, by means of fibrin cloths as temporary barriers (Sinno and Prakash, 2013). *Inflammation* (form 24 h to 4–6 days) is mediated by neutrophils and macrophages (Broughton et al., 2006), that sweep the wound bed from foreign particles and tissue debris. Cytokines and enzymes are released to stimulate fibroblasts and myofibroblasts (Das and Baker, 2016), while the wound exudate provides the essential moisture for the recovery. In the *proliferation* phase epithelialization occurs and newly formed granulation tissue begins to fill the wound area, producing new extracellular matrix (ECM). Finally, during the *remodeling* phase, collagen-based cross-linking is responsible for a tight 3D network formation, increasing the tensile strength of the new tissue (Sinno and Prakash, 2013).

Given the multiple mechanisms involved in the skin wound healing and the interplay of several external factors, the choice of suitable dressing materials is compelling. Specifically, for biodegradable natural materials, their degradation needs to follow the dynamics of the wound repair, guaranteeing the physiological healing evolution, and releasing active principles when needed. At last but not least, proper consideration should be put onto the environmental sustainability of these biomaterials, in terms of green chemistry fabrication approaches, and complete biodegradation without harmful by-products. While numerous reviews on traditional wound dressing biomaterials have been extensively published (Sell et al., 2010; Mogoşanu and Grumezescu, 2014; Norouzi et al., 2015; Mele, 2016), in this mini-review we will focus our attention on the most recent naturally-derived, active systems, pursuing the quest for an environmentally sustainable wound management.

## Mimicking nature as a therapeutic strategy

Successful wound management relies on understanding the healing process combined with a know-how on the properties of the various dressing materials available. Principal purpose of any wound treatment is to maximize the treatment efficiency (Weller and Sussman, 2006). Currently, standard care procedures consist of swabbing the infection, cleaning the wound bed from tissue debris, and applying the dressing (Dreifke et al., 2015). In case of extended skin lesions, the use of split-thickness skin autografts or allografts might be required, carrying safety issues related to disease transmission and immune rejection.

An ideal dressing should remove excessive exudate to avoid tissue maceration and promote autolytic debridement, while keeping moisture, adequate oxygen and water vapor permeability within the wound. It should be adhesive and flexible, to favor mechanical compliance to the patient body and ease the application/removal. Deliverable bioactive compounds, such as antibiotics, essential oils, and natural antioxidants, stimulate the dressing interaction with the wound microenvironment and further enhance the therapeutic action via antimicrobial, antifungal, and antiseptic activities (Pereira et al., 2013). A number of fabrication techniques, such as film-casting, electrospinning, self-assembly, freeze-drying, emulsions, microsphere injection, have been employed to produce wound dressings, either based on synthetic macromolecules or on materials of natural origin (Sell et al., 2007, 2010; Wei and Ma, 2008; Huang and Fu, 2010; Zhong et al., 2010; Rieger et al., 2013; Mogoşanu and Grumezescu, 2014; Norouzi et al., 2015; Liakos et al., 2016; Mele, 2016).

**Figure 1:**
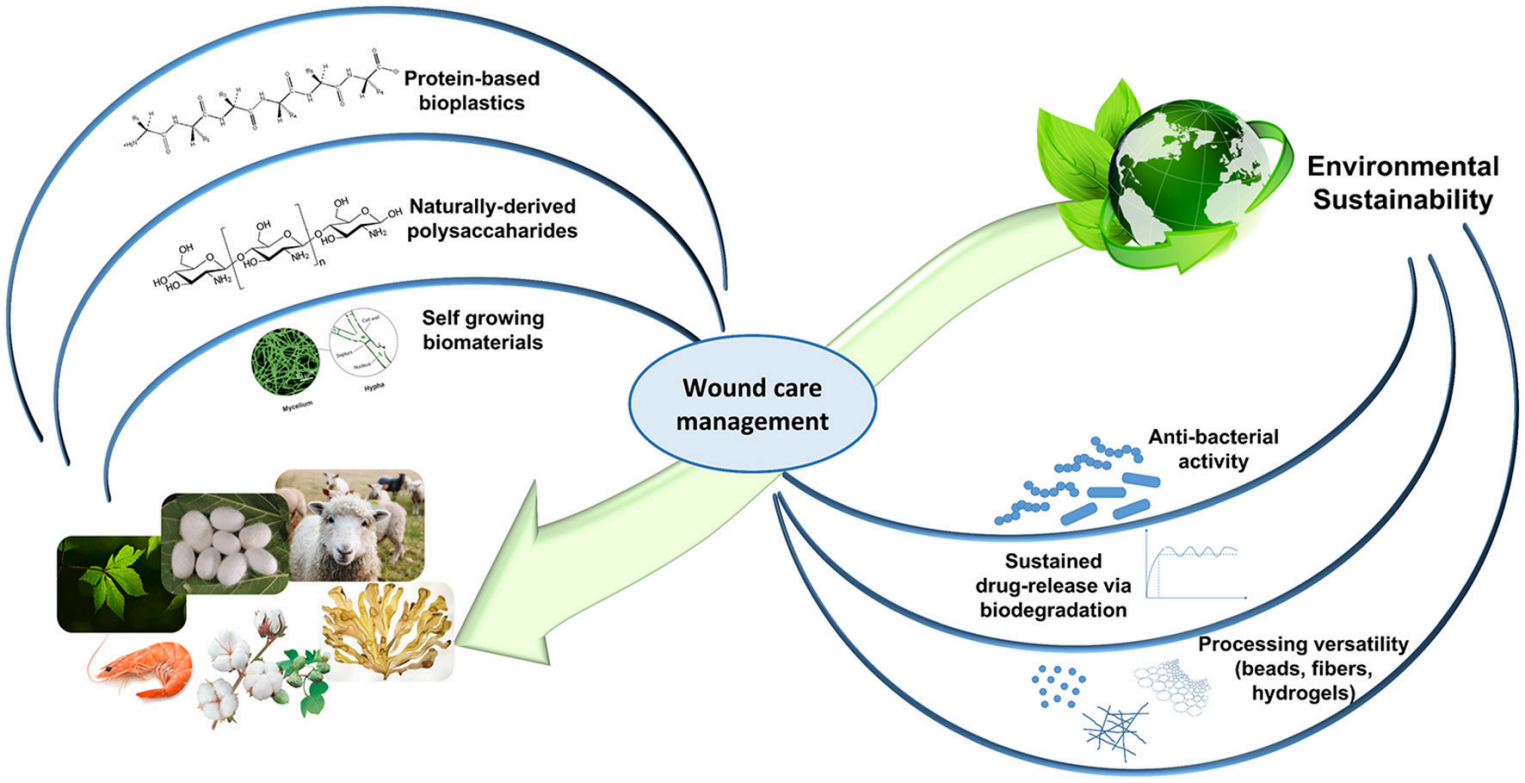
The circularity concept of Nature-mimicking for an environmentally-friendly wound healthcare. Self-growing biomaterials panel is adapted from Haneef et al. (2017). This material is licensed under the Creative Commons Attribution 4.0 International Public License (https://creativecommons.org/licenses/by/4.0/legalcode).

Recently, natural biopolymers have largely attracted the scientific community interest. On top of their notable biocompatibility and biodegradability, natural occurring proteins and polysaccharides allow to achieve the highest level of biomimicry, recapitulating the native ECM biological and physico-chemical features. Further architectural resemblance can be obtained with an appropriate processing (e.g., nanofibers, sponge-like hydrogels; Huang and Fu, 2010; Mogoşanu and Grumezescu, 2014; Liakos et al., 2015; Mele, 2016). Despite their batch-to-batch variations in terms of mechanical properties and their rather limited shelf-life, naturally-derived biopolymers confer ECM support (collagen, gelatin, hyaluronic acid), present cell-recognition domains and biomolecule binding sites (RGD and LDV sequences in silk fibroin and keratin), and may possess inherent antibacterial and anti-inflammatory properties (chitosan, alginate). Moreover, in the past decades, clinical understanding advancements have directed significant exploitation of natural materials in clinical trials (Vyas and Vasconez, 2014; Dhivya et al., 2015). By looking into our natural surroundings and by re-using some of the discarded natural resources, several functional biomaterials can be easily identified and implemented for promising wound healing applications, with a reduced impact on the environment (Figure 1).

## Protein-based biopolymers

### Collagen and gelatin

**Figure 2:**
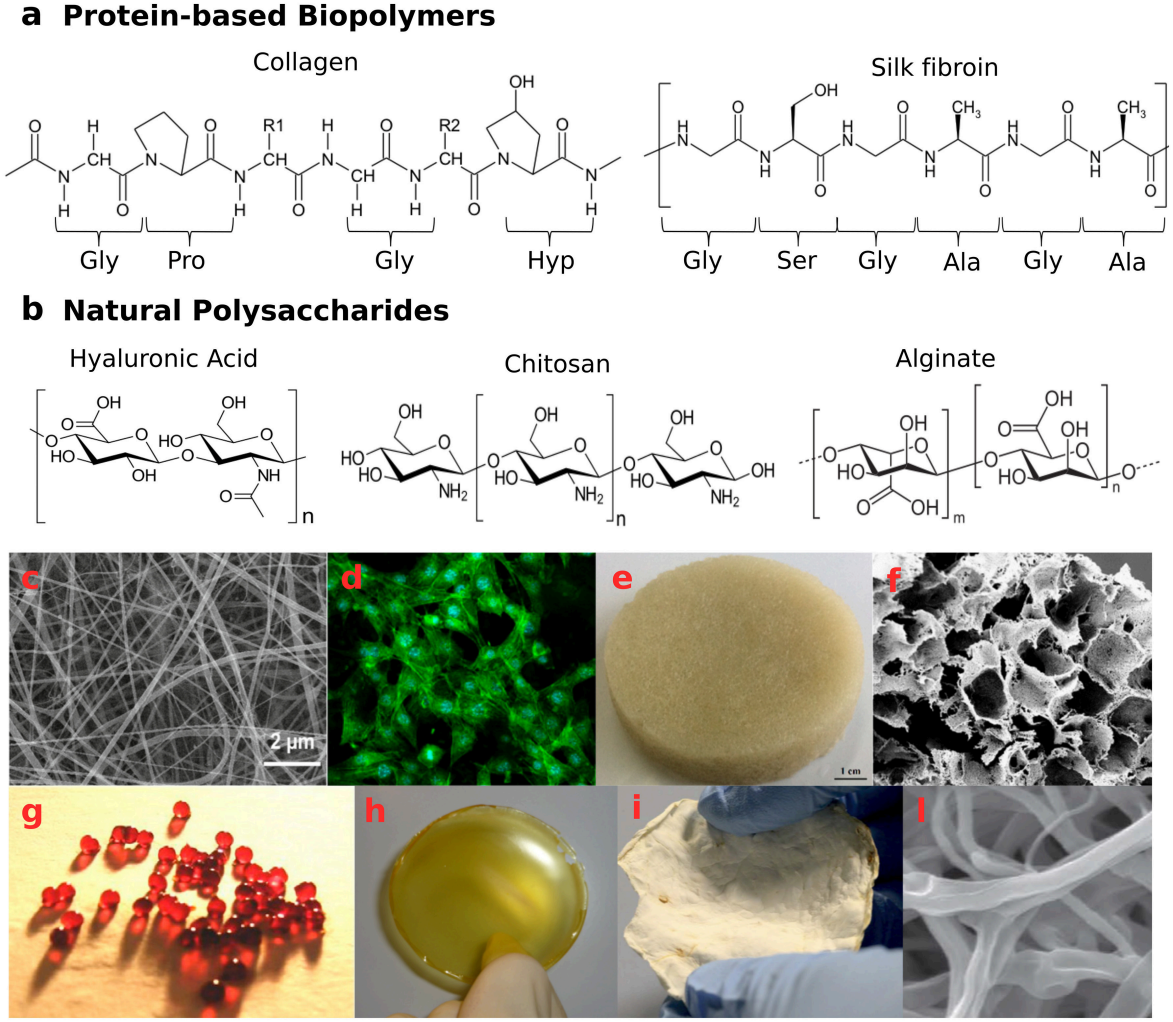
Naturally-derived biopolymer-based structures with potential application as wound healing systems. **(a)** examples of protein-based biopolymers primary structures—aminoacidic sequence of collagen type I molecules and aminoacidic sequence of silk fibroin molecules: Gly, glycine; Ala, alanine; Pro, proline; Ser, serine; Hyp, hydroxyproline; **(b)** natural polysaccharide structures—hyaluronic acid, chitosan, and alginate; **(c,d)** Biocompatible silk/parsley electrospun fibers (average diameter 50 nm) able to grow NIH3T3 fibroblast cells adapted with permission from Guzman-Puyol et al. (2016) Copyright^©^2016 American Chemical Society; **(e,f)** wool keratin sponges, reprinted from Patrucco et al. (2016) Copyright^©^2016 with permission from Elsevier; **(g)** calcium cross-linked alginate beads and **(h)** film incorporating antiseptic PVPI complex, reprinted from Liakos et al. (2013) Copyright^©^2013 with permission from Elsevier; **(i,l)** mycelia material from *P. ostreatus* after 20 days of growth on potato-dextrose broth and cellulose, presenting a 3D network of hyphae. Panels **(i,l)** are adapted from Haneef et al. (2017). This material is licensed under the Creative Commons Attribution 4.0 International Public License (https://creativecommons.org/licenses/by/4.0/legalcode).

Collagen is the most abundant animal protein, which provides mechanical strength to tissues and stimulates cell-adhesion and proliferation (Neel et al., 2013; An et al., 2016). Twenty-nine different types of collagen have been identified, displaying a triple-helical tertiary structure of polypeptide sequences (Figure 2a), but only a few are used in the production of collagen-based biomaterials. As animal-derived proteins may be responsible for allergic reactions and pathogen transmissions (Koide, 2007), an alternative is constituted by collagen from heterologous expression in mammalian, insect and yeast cells (Olsen et al., 2003), or produced by *Escherichia coli* (Pinkas et al., 2011). High biocompatibility and biodegradability by endogenous collagenases make collagen ideal for biomedical applications (Parenteau-Bareil et al., 2010; Chattopadhyay and Raines, 2014). During wound healing, fibroblasts produce collagen molecules that aggregate to form fibrils with diameter in the range of 10–500 nm. This fibrous network facilitates cell migration to the wounded site, actively supporting tissue repair (Baum and Arpey, 2005).

Thanks to a facile chemical functionalization of the protein structure, various dressing architectures have been exploited. Collagen-based wound dressings, either in forms of hydrogels, electrospun fibers, or nanocrystal-containing scaffolds, have been applied to cover burn wounds, treat ulcers (Ghica et al., 2017; Guo et al., 2017; Bhowmick et al., 2018; Yoon et al., 2018), reduce tissue contraction and scarring, and increase epithelialization rate (Powell et al., 2008). Collagen sponges and fibrous membranes were found particularly promising, due to their wet-strength that allows suturing to soft tissues and provides a template for new tissue growth. Composites with other natural materials, such as dextran, chitosan, hyaluronic acid, and alginate (Karri et al., 2016; Ghica et al., 2017; Wei et al., 2018) or constructs based on collagen and synthetic biopolymers, such as poly-α-hydroxyl esters (Hall Barrientos et al., 2017; Albright et al., 2018; Bhowmick et al., 2018) have been extensively exploited. Moreover, acetylated, succinylated, methylated, or biotinylathed collagen have been used to immobilize therapeutic enzymes or growth factors and to control drug delivery (Lima et al., 2015; Mele, 2016; Qu et al., 2018; Zhu et al., 2018). Albright and coworkers (Albright et al., 2018) proposed a multi-structured nanofibrous dressing, composed of poly-ε-caprolactone/collagen electrospun matrix, loaded with transforming growth factor TGF-β1 and modified with polypeptide-based nanocarriers incorporating tannic acid and gentamicin. The multifunctional platform showed anti-bacterial and anti-inflammatory properties, while retaining a favorable topography for cell proliferation, thus accelerating healing and wound closure. A similar construct was proposed by Karri et al. (2016), where a composite scaffold of collagen and alginate was impregnated with curcumin-loaded chitosan nanoparticles to obtain an all-natural wound dressing.

A collagen-derivative with promising biomedical values is gelatin. Gelatin is obtained by an incomplete denaturalization of collagen extracted from connective tissues, skin, and boiling bones (Jaipan et al., 2017). It has been employed to fabricate strong hydrogel-like membranes (Thanusha et al., 2018), microspheres (Thyagarajan et al., 2017), sponges, and electrospun mats (Chen et al., 2016), for dermal tissue applications and to treat severe burn wounds. Various combinations of gelatin and modified chitosan have been proposed (Han et al., 2014; Agarwal et al., 2016), as well as blends with poly-vinyl alcohol based via enzymatic crosslinking, to support fibroblast culture and proliferation (Hago and Li, 2013).

Despite their rather extensive usage as biomaterials for scaffold design, collagen and gelatin remain sustainable materials with highly engineering potential yet unexplored (Hall Barrientos et al., 2017; Golser et al., 2018).

### Silk fibroin

Silks are proteins produced in the epithelial cells of specialized glands of various arthropods, such as spiders and silkworms. The secreted silk fibers present a highly repetitive sequence, consisting mainly of glycine (43%), alanine (30%), and serine (12%)—[GAGSGA]_n_ motifs, arranged in β-sheets regions embedded in an amorphous matrix (Chutipakdeevong et al., 2013; Reimers et al., 2015), which confer high toughness and elasticity (Figure 2a). Regarding more specifically silk from the cocoons of *Bombyx mori* silkworms, two kinds of proteins are its major components: the fibroin and the sericin. The fibroins are composed of three types of protein fibers: 350 kDa-heavy chain, 30 kDa-glycoprotein, and 25 kDa-light chain, the latter conferring hydrophilicity, water uptake ability and cell adhesion properties. Light and heavy chains are connected by disulfide bonds, while the glue protein sericin coats the silk fibers. Thanks to high mechanical resistance, enzymatic-driven biodegradability and favorable cell attachment, silk fibroins have been successfully exploited for skin tissue engineering and wound healing applications.

*Bombyx mori* silkworm-derived fibroins are obtained from cocoon and separated from sericin by degumming in alkaline boiling water and following solubilization in hot LiBr solution (Reimers et al., 2015). Regenerated silk water soluble form, silk I, can be converted into insoluble silk II, by modifying the α-helical chain arrangements into β-sheets via alcohol treatment or water vapor annealing (WVA; Min et al., 2006; Wharram et al., 2010; Hu et al., 2011). In fact, by controlling the protein secondary structure, fibroin scaffolds' biodegradation can be properly tuned, in order to modulate the release of bioactive molecules (Hofmann et al., 2006), such as antibiotics (Pritchard et al., 2013; Chouhan et al., 2017), growth factors (Schneider et al., 2009; Chouhan et al., 2017; Pignatelli et al., 2018), and anti-oxidant compounds (Fan et al., 2012; Sheng et al., 2013; Lin et al., 2016). In their study, Pignatelli and coworkers (Pignatelli et al., 2018) encapsulated human platelet lysate into electrospun silk-PEO patches, to prolong the growth factor shelf life and ease its handling during wound management. By changing the crystallinity degree of the fibrous matrices via WVA from 21 to 35 or 44%, the 24-h drug release drastically decreased from 100 to 80 and 46%, respectively.

Proteins' versatile nature allows for a plethora of processing techniques, with the consequent fabrication of multiple scaffold morphologies, such as films (Srivastava et al., 2015), foams and sponges (Roh et al., 2006), gels, and fibrous matrices. In the last decade, electrospun fibroin has been extensively proposed for the design of anti-bacterial, anti-inflammatory and anti-oxidant patches (Lin et al., 2016; Selvaraj and Fathima, 2017; Yang et al., 2017). To ease a water-based electrospinning process, silk has been processed either in combination with natural polymers, such as cellulose (Guzman-Puyol et al., 2016; Figures 2c,d), gelatin (Shan et al., 2015), sericin (Hang et al., 2012), chitosan (Cai et al., 2010), alginate (Roh et al., 2006), elastin (Zhu et al., 2016), and hyaluronic acid (Yan et al., 2013), or mixed with synthetic materials, such as polyethylene oxide (Schneider et al., 2009; Wharram et al., 2010; Chutipakdeevong et al., 2013), polyvinyl alcohol (PVA; Chouhan et al., 2017), and poly-hydroxy esters (Lian et al., 2014; Shahverdi et al., 2014; Suganya et al., 2014; Shanmugam and Sundaramoorthy, 2015). Silk/PVA mats loaded with Ciprofloxacin and epidermal growth factors (Chouhan et al., 2017) enhanced human dermal fibroblasts and keratinocytes proliferation *in vitro*, and favored re-epithelization, mature collagen deposition and complete wound closure at 14 days in a *in vivo* wound healing rabbit model. In a different work, Ju and coworkers (Ju et al., 2016) investigated the intrinsic anti-inflammatory effects of a porous fibroin/PEO electrospun nanomatrix in a mice burn-model, observing downregulation of pro-inflammatory cytokines IL-1α and IL-6.

### Keratin

Keratins (Ker) are the most abundant group of insoluble and filament-forming proteins produced in epithelial cells of mammals, birds, reptiles, and humans. As structural components of wool, nails, horn, feathers, and hair, they exploit mechanical support and protective functions against the environment (Reichl, 2009; Wang et al., 2016). Keratins present a complex intermediate filament (IF)-matrix hierarchical structure and are categorized according to the polypeptide chain secondary assembly. α-Ker (40–68 kDa) comprise α-helices arranged in coiled-coil heterodimers to form 7-nm IF, while β-Ker (10–22 kDa) consist of packed β-sheets disposed in 3-nm IF. The high-sulfur containing matrix (γ-Ker, below 10 kDa), rich in cysteine, tyrosine, glycine and phenylalanine residues, present a globular assembly (Dowling et al., 1986; Fraser et al., 1986; Steinert and Marekov, 1993; Rouse and Van Dyke, 2010; Wang et al., 2016). The secondary structure of keratinous materials largely affects their mechanical resistance, solubility, and hydration sensitivity (Wang et al., 2016). Tons of Ker-containing biomasses are produced every year, from meat and poultry market, wool industry and hair salons, leading to continuous accumulation of wastes in the ecosystem. The challenges associated with this waste disposal have been considered by the European Parliament and Council regulation EC 1774/2002 (Sharma and Gupta, 2016). Due to the presence of strong disulfide and H-bonds, keratin extraction from biomasses involves rather complicated methods, such as microbial and enzymatic hydrolysis, mechanical treatments, or chemical protocols with alkali, reducing agents or ionic liquids (Yamauchi et al., 2003; Ozaki et al., 2014; Sharma and Gupta, 2016; Shavandi et al., 2017).

However, thanks to its biocompatibility, biodegradability, and hemostatic properties, keratin constitutes a potential green secondary raw material for wound healing, tissue repair, drug delivery, and cosmetics applications (Sharma and Gupta, 2016; Arslan et al., 2017; Shavandi et al., 2017). Since its earliest documented use in medicinal applications (China, sixteenth century; Rouse and Van Dyke, 2010), in the past decades several Ker-based biomaterials have been proposed, given the ability of this biopolymer to self-assembly into 3D networks favorable for cell infiltration, and its intrinsic bioactivity for the presence of cell binding motifs (such as EDS, LDV, and RGD; Rouse and Van Dyke, 2010). Keratin extracted from chicken feathers, wool and human hair have been processed in films (Yamauchi et al., 2003; Fujii and Ide, 2004; Tonin et al., 2007; Reichl, 2009; Cui et al., 2013), sponge-like and hydrogel-like scaffolds for tissue engineering and wound healing (Figures 2e,f; Tachibana et al., 2002; Verma et al., 2008; Hill et al., 2010; Saul et al., 2011; Richter et al., 2012; Wang et al., 2012; Xu et al., 2013; Patrucco et al., 2016; Singaravelu et al., 2016). Electrospun fibers have also been obtained in combination with PEO (Aluigi et al., 2007, 2008; Fan et al., 2016; Ma et al., 2017), PVA (Choi et al., 2015; He et al., 2017; Wang et al., 2017), fibroin (Zoccola et al., 2008; Yen et al., 2016), poly-caprolactone (Boakye et al., 2015; Edwards et al., 2015; Li et al., 2016; Zhu et al., 2017), poly(3-hydroxybutyrate-co-3-hydroxyvalerate) (Yuan et al., 2015), chitosan (Singaravelu et al., 2016), and gelatin (Yao et al., 2017).

## Naturally-derived polysaccharides

### Hyaluronic acid

Hyaluronic acid (HA) is a non-immunogenic polysaccharide consisting of glucuronic acid and N-acetyl-D-glucosamine units (Figure 2b). This glycosaminoglycan is one of the main components of the connective tissue in mammals (Mele, 2016). Due to its hygroscopic nature, HA has been used to prepare hydrogel-like constructs, to support keratinocyte migration and angiogenesis, and promote a scar-free wound healing (Mogoşanu and Grumezescu, 2014; Dreifke et al., 2015). The molecular weight (MW) plays a key role in the process (Tolg et al., 2014): low MWHA degradation products were found to be pro-inflammatory (Campo et al., 2010; Dreifke et al., 2015), while high MWHA appeared to inhibit nutrient supply. Interestingly, medium MWHA (100–300 kDa) showed enhanced wound closure capability through up-regulation of adhesion molecules (Ghazi et al., 2012). Moreover, the hydrophilicity of the HA chains allows the 3D network swelling and the consequent gradual release of encapsulated active compounds, making this biomaterial suitable as drug delivery platform (Maeda et al., 2014). HA-based electrospun fibers, either pure or in combination with other biomacromolecules (Xu et al., 2009; Hsu et al., 2010; Uppal et al., 2011; Dogan et al., 2016), have been proposed for tunable degradation and sustained release *in vitro* and *in vivo*.

### Chitosan

Chitosan (CS), a deacetylated chitin-derivative found in the exoskeletons and shells of crustaceans, is a linear polysaccharide consisting of β(1-4)-D-glucosamine and N-acetyl-D-glucosamine groups randomly distributed (Figure 2b). Owing to its intrinsic antifungal, antibacterial, hemostatic, and muco-adhesive properties, chitosan has been widely exploited in the biomedical field for wound and burn treatments (Dash et al., 2011; Croisier and Jérôme, 2013; Norouzi et al., 2015; Zhao et al., 2015). Several dressing architectures have been proposed: CS-*Aloe vera* membranes (Wani et al., 2010), thyme oil-CS films (Altiok et al., 2010), CS-gelatin sponges (He et al., 2007), CS-silk hydrogels (Silva et al., 2012), CS-cellulose films (Niyas Ahamed and Sastry, 2011; Romano et al., 2015a), cinnamon oil-CS/polyethylene oxide nanofibers (Rieger and Schiffman, 2014), and CS/poly(3-hydroxybutyrate-*co*-3-hydroxyvalerate) scaffolds (Veleirinho et al., 2012). In addition, water-soluble derivatives, such as carboxymethyl-CS and methacrylate glycol CS have been synthetized and investigated for wound healing applications (Romano et al., 2015b).

### Alginate

Alginate (Alg) is a linear co-polymer of β-D-Mannuronic acid and α-L-Glucuronic acid (Figure 2b). This polysaccharide is mostly abundant in *Brown Algae* or produced by some bacteria (Khan and Ahmad, 2013). It is highly hydrophilic, biocompatible, and able to absorb wound exudate, maintaining a moist microenvironment (Chiu et al., 2008). The combination of alginate with antimicrobial and enzymatic components can promote elimination of necrotic tissues and microbial bodies, while the polysaccharide base can stimulate reparative wound processes (Patel et al., 2007). Alginate dressings are also useful as delivery platforms, in order to provide a controlled release of therapeutic substances to exuding wounds (e.g., pain-relieving, antibacterial, and anti-inflammatory agents; Maver et al., 2015; Szekalska et al., 2016; Setti et al., 2018). Biodegradable Na-Alg/PVPI (povidone iodine complex) films and Ca-Alg/PVPI beads have displayed antimicrobial and antifungal activities (Liakos et al., 2013; Figures 2g,h). Moreover, Na-Alg/PVPI films have shown to reduce the inflammatory response and accelerate the wound healing providing a controlled release of PVPI (Summa et al., 2018). To treat UV-induced skin burns, instead, electrospun nanofibers loaded with lavender essential oil have been used: the composite mats exhibited antibacterial and anti-inflammatory properties, being able to reduce the production of pro-inflammatory cytokines both *in vitro* and *in vivo* (Hajiali et al., 2016).

## Self-growing mycelium-based biomaterials

Mycelium, the fungi vegetative part, comprises a network of filamentous hyphae, which penetrate the substrate. Hyphae are tubular structures of micrometric diameter, composed of aligned and elongated cells, separated by walls, called septa (Figures 2i,l). A continuous cell wall protects the hyphae and confers mechanical strength and shape to the mycelium (Haneef et al., 2017; Jones et al., 2017). Being constituted of chitin, chitosan, glucans, mannoproteins, and glycoproteins (Synytsya and Novák, 2013), the cell wall is a biopolymer composite that prevents the hyphae from collapsing during their sprouting and movement (Cairney, 2005).

Peculiarity of these living, self-growing composites is the possibility to tune the physico-chemical properties during their growth phase, reducing sophisticated processing and by-product formation, while allowing for ready-to-use systems. Throughout its dynamic growth, the mycelium “senses” the substrate and responds to the surrounding, depending on edaphic conditions, substrate pH and composition, or the presence of other living organisms (Krull et al., 2013). A polarized extension of the cell wall occurs at the apical region of the hyphae, as the mycelium secretes a variety of enzymes, hydrolyzes the substrate and absorbs the solubilized nutrients. By properly exploiting different feeding substrates for hyphae digestion, the resulting properties of the interwoven fibrous mycelium material can be efficiently tailored. Type and amount of absorbed nutrients may affect mycelia growth rate, extension and biological activity (Frimpong-Manso et al., 2011; Da Silva et al., 2012; Larsen, 2015; Anderson and Cairney, 2018). Similarly, culture conditions and feeding substrate highly influence the final chemical composition (Krull et al., 2013; Haneef et al., 2017), either stimulating plasticizer biosynthesis (lipids and small glycoproteins) or promoting rigid macromolecule production (chitin, β-D-glucans; Synytsya and Novák, 2013). In this regard, mycelia can be considered as 3D smart, micro-reactors, able to bio-convert various agro-residues into enzymes, polysaccharides, and bioactive metabolites (Vassilev et al., 1995; Krull et al., 2013; Vamanu, 2014; Yan et al., 2014; Salati et al., 2017), with potential bio-pharmaceutical and neutraceutical relevance.

In the past, mycelia-derived scaffolds have been exploited (Su et al., 1999, 2005; Hung et al., 2001). Su et al. (1997) developed a filament-structured membrane from the residue of *Ganoderma tsugae*, called *Sacchachitin*, composed of β-1,3-glucan (60%), and N-acetylglucosamine (40%), to be used as skin substitute. The new biomaterial demonstrated wound healing potential *in vivo*, by promoting fibroblast proliferation and migration. In a following study (Su et al., 2005), the *Sacchachitin* membranes appeared to boost keratinocytes proliferation and prevent metalloproteinase-related ECM degradation, contributing to accelerate the healing process of a chronic wound *in vivo* model. Furthermore, a micronized *Sacchachitin* nanogel has been investigated to treat superficial chemical corneal burns *in vivo* (Chen et al., 2012), while the anti-oxidant and immuno-modulating effects of extracts from mycelia of some medicinal fungi have been investigated for skin aging (Kim et al., 2014), dermatitis (Hwang et al., 2012), and UV-protection (Nanbu et al., 2011; Bae et al., 2012), suggesting a promising mycelia biological value yet unexplored.

## Conclusions - a quest for an environmentally sustainable wound management

Environmental sustainability has nowadays become an imperative issue to front, in an effort to balance both the industrial productivity and the planet ability to generate resources, with the neutralization of wastes and the mitigation of polluting processes. Material and energy consumption related to the healthcare industry, ranging from complex material manufacturing processes, to drug packaging, to high-volume medical wastes, might heavily contribute to increase the overall pollution, with an unsought negative impact on the human health (Jameton and Pierce, 2001). Nature itself can be of inspiration to develop cost-competitive, low-energy consumption and fully biodegradable materials, presenting greater environmental sustainability. The increasing interest of the scientific community in the use of either protein-based or polysaccharide-derived dressings is striking, and it reflects the growing perspective of giving back what we borrowed from Nature. In this regard, self-growing mycelia, which are biocomposites constituted of both proteins and polysaccharides, can represent a smart strategy to fabricate healthcare products of the future, as economically and environmentally valid alternatives to synthetic materials. In conclusion, naturally derived biopolymers provide a versatile, multifunctional, and tunable platform to design appropriate extracellular environments, able to actively contrast the onset of infections and inflammations, while promoting tissue regeneration, and scar remodeling.

## Author contributions

GS and RB conceived and wrote the main manuscript text. AA edited and reviewed the whole manuscript.

### Conflict of interest statement

The authors declare that the research was conducted in the absence of any commercial or financial relationships that could be construed as a potential conflict of interest.
